# Supplementary material for: Phosphorylation-Dependent SERS Readout for Activity Assay of Protein Kinase A in Cell Extracts
Source: Nanomaterials (Basel). 2020 Mar 22;10(3):575. doi: 10.3390/nano10030575 (PMC7153394; doi:10.3390/nano10030575)
Supplement: Supplementary file 1 [file nanomaterials-10-00575-s001.pdf]

Supplementary Materials

Phosphorylation-Dependent SERS Readout for Activity Assay of Protein Kinase A in Cell Extracts

Renyong Liu [1], Chenggen Xie [1], Yehan Yan [1], Lin Hu [2,*], Suhua Wang [3,4], Khalid A. Alamry [4], Hadi M. Marwani [4] and Lijuan Chen [1,*]

1 Key Laboratory of Biomimetic Sensor and Detecting Technology of Anhui Province, School of Materials and Chemical Engineering, West Anhui University, Lu'an, Anhui 237012, China; ahlry@mail.ustc.edu.cn (R.L.); cgxie@wxc.edu.cn (C.X.); yanyh@mail.ustc.edu.cn (Y.Y.)

2 Anhui Province Key Laboratory of Condensed Matter Physics at Extreme Conditions, High Magnetic Field Laboratory, Chinese Academy of Sciences, Hefei, Anhui 230031, China

3 College of Environmental Science and Engineering, North China Electirc Power University, Bejing 102205, China; wangsuhua@ncepu.edu.cn (S.W.)

4 Chemistry Department, Faculty of Science, King Abdulaziz University, Jeddah 21589, Saudi Arabia; k_alamry@yahoo.com (K.A.A.); hmarwani@kau.edu.sa (H.M.M.)

* Correspondence: hulingg@mail.ustc.edu.cn (L.H.); lijuanch@mail.ustc.edu.cn (L.C.); Tel.: +86-0551-65591149 (L.H.); +86-0564-3305690 (L.C.)

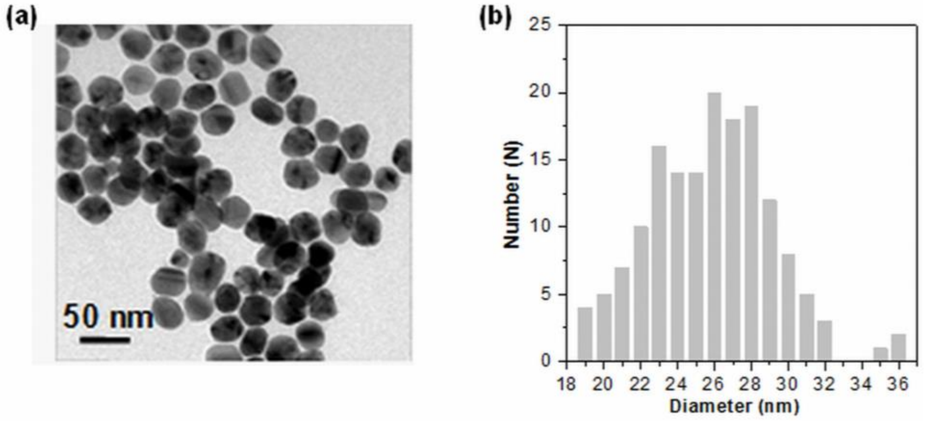

Figure S1. (a) TEM image of spermine-functionalized AgNPs in aqueous solution. (b) The size distribution of spermine-functionalized AgNPs determined by TEM examination (N = 158).

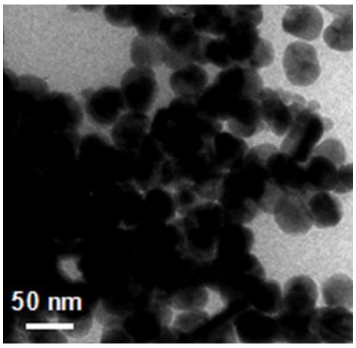

Figure S2. TEM image of the aggregated AgNPs in Tris-HCl buffer (50 mM, pH 7.5).

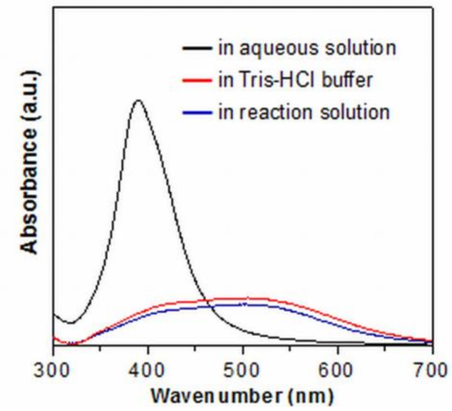

Figure S3. UV-vis spectra of AgNPs in aqueous solution, Tris-HCl buffer and PKA reaction solution, respectively.

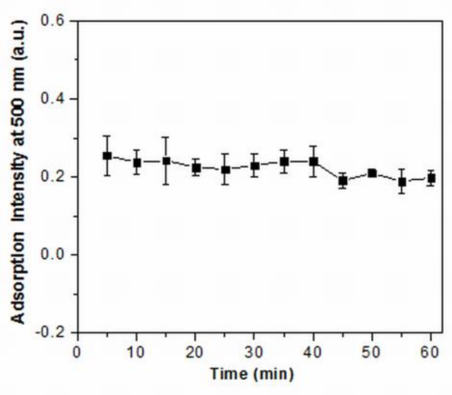

Figure S4. The absorption intensity of aggregated AgNPs (~500 nm) as a function of the incubation time.

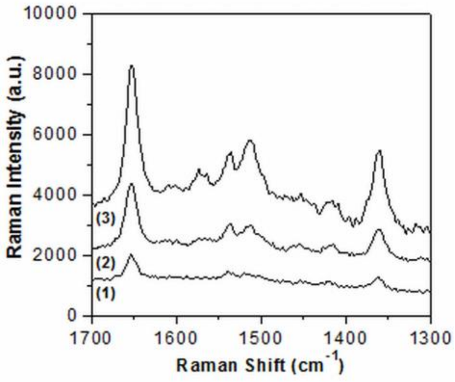

Figure S5. SERS spectra obtained from HeLa cell extracts (1) without or with the stimulation of (2) 10 μM forskolin/20 μM IBMX and (3) 25 μM forskolin/50 μM IBMX, respectively.
